# Sex Ratio Bias and Extinction Risk in an Isolated Population of Tuatara (*Sphenodon punctatus*)

**DOI:** 10.1371/journal.pone.0094214

**Published:** 2014-04-08

**Authors:** Kristine L. Grayson, Nicola J. Mitchell, Joanne M. Monks, Susan N. Keall, Joanna N. Wilson, Nicola J. Nelson

**Affiliations:** 1 Allan Wilson Centre for Molecular Ecology and Evolution, School of Biological Sciences, Victoria University of Wellington, Wellington, New Zealand; 2 Centre for Evolutionary Biology, School of Animal Biology, The University of Western Australia, Crawley, W.A., Australia; 3 Science and Capability Group, Department of Conservation, Christchurch, New Zealand; Smithsonian Conservation Biology Institute, United States of America

## Abstract

Understanding the mechanisms underlying population declines is critical for preventing the extinction of endangered populations. Positive feedbacks can hasten the process of collapse and create an ‘extinction vortex,’ particularly in small, isolated populations. We provide a case study of a male-biased sex ratio creating the conditions for extinction in a natural population of tuatara (*Sphenodon punctatus*) on North Brother Island in the Cook Strait of New Zealand. We combine data from long term mark-recapture surveys, updated model estimates of hatchling sex ratio, and population viability modeling to measure the impacts of sex ratio skew. Results from the mark-recapture surveys show an increasing decline in the percentage of females in the adult tuatara population. Our monitoring reveals compounding impacts on female fitness through reductions in female body condition, fecundity, and survival as the male-bias in the population has increased. Additionally, we find that current nest temperatures are likely to result in more male than female hatchlings, owing to the pattern of temperature-dependent sex determination in tuatara where males hatch at warmer temperatures. Anthropogenic climate change worsens the situation for this isolated population, as projected temperature increases for New Zealand are expected to further skew the hatchling sex ratio towards males. Population viability models predict that without management intervention or an evolutionary response, the population will ultimately become entirely comprised of males and functionally extinct. Our study demonstrates that sex ratio bias can be an underappreciated threat to population viability, particularly in populations of long-lived organisms that appear numerically stable.

## Introduction

Biased population sex ratios are a recognized problem for the conservation and management of vulnerable populations. Female-biased populations, which can result in mate limitation at extreme ratios, are usually less of a concern for conservation managers because increased numbers of females can boost reproduction and increase population growth rate [1]. An overabundance of males can be more problematic, as higher male-male competition and the production of fewer offspring are all detrimental to population growth and viability [2]–[4].

In small populations biased sex ratios can arise through demographic stochasticity, where low reproductive rates can result in the over-production of one sex due to chance [5]. Adult sex ratios can become skewed through sex-biased mortality [6] or harvesting [7]. Hatchling sex ratios can also be influenced by differential survival of each sex, or by environmental conditions. For example, maternal condition in some birds and mammals can influence brood sex ratios [8], [9] and cases of population-wide food limitation or supplementation have resulted in skewed hatchling sex ratios [10], [11]. In species with environmental sex determination (ESD), offspring sex develops based on the conditions in the immediate environment such as habitat, nutrition, photoperiod, or population density [12]. A form of ESD prevalent in reptiles is temperature-dependent sex determination (TSD), where incubation temperature during a specific window of embryonic development determines sex [13].

In reptiles with TSD, changes in habitat or climate have the potential to shift the sex ratio of hatchlings produced from nests and rapid directional changes in temperature can result in biased offspring sex ratios [14]. Several studies have shown that unusually warm years produce hatchling sex ratios skewed towards the sex produced at the upper end of the TSD pattern [15]–[17]. Changes in climate can impact hatchling sex ratios through direct effects on incubation temperature and indirect effects on the phenology and duration of breeding seasons. Most species of reptiles with TSD have patterns that produce females at warmer temperatures (male→female or female→male→female as incubation temperature increases)[18]. Reptiles that have the rare female→male pattern of sex determination are most at risk from demographic instability through an over-production of males under climate change [14]. However, environmental changes that result in the production of a single sex, regardless of the TSD pattern, have obvious implications for population viability.

Regardless of the mechanism that creates a sex ratio bias within a population, increased numbers of males can result in behaviors that impose additional fitness costs on females, such as mating harassment [19], [20]. Recent work has shown that a male-biased sex ratio can create positive feedbacks that further the decline of female numbers and increase the risk of population extinction [21], [22]. Gilpin and Soulé [23] identified demographic variation as one of four ‘extinction vortexes’ or mechanisms that increase extinction risk as a population becomes smaller. Compared to other risks to small populations, such as inbreeding depression [24] and Allee effects [25], studies examining the link between sex ratio bias and extinction risk are limited, particularly in natural populations.

In this study we examine the impacts of male sex ratio bias in a natural population of an iconic New Zealand reptile, the tuatara (*Sphenodon punctatus*). North Brother Island is a small conservation reserve hosting a well-studied tuatara population, where a male-bias became apparent in the late-1990s [26]. In this study we examine ten more years of data and find that the male-bias in the population sex ratio has continued to increase. Here we explore the mechanisms behind this change and examine the consequences for population viability. We demonstrate that three compounding factors are contributing to the skew in adult sex ratio and are likely to result in functional extinction of the population: 1) decreasing body condition of adults, 2) reduced adult survival, and 3) shifts in hatchling sex ratio due to climate change.

## Methods

### Study species and site

**Figure 1:**
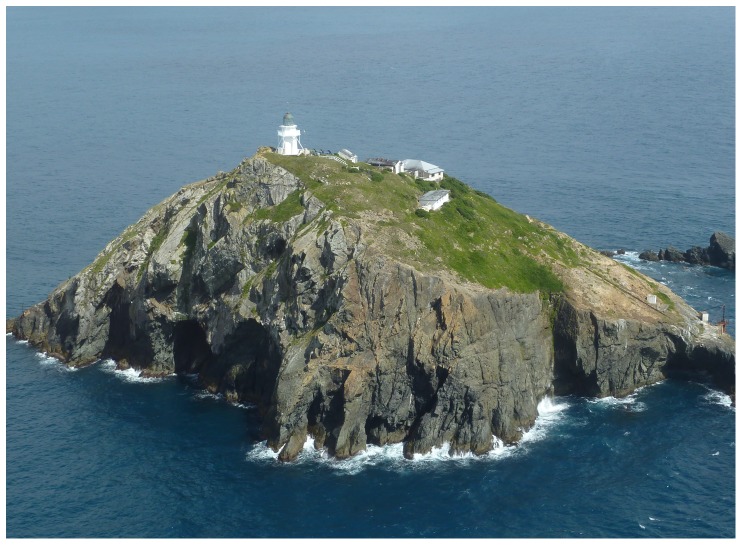
North Brother Island in the Cook Strait of Zealand. A rocky, 4 hectare wildlife sanctuary and lighthouse station that rises to 66

Tuatara are long-lived Sphenodontian reptiles endemic to New Zealand and are the only surviving species of the order Rhynchocephalia. They are nocturnal, adapted to cool climates, and their life history is characterized by a long maturation time (15–20 years) and large intervals between breeding events in females (typically 2–5 years) [27]. Tuatara have TSD pattern Type IB, where eggs from warmer nests produce male hatchlings while cooler nests produce females [28]. Once widespread across the two main islands of New Zealand, tuatara are now limited to approximately 40 populations on small offshore islands due to the introduction of mammalian predators and habitat modification [29]. North Brother Island is a 4 ha wildlife sanctuary in the Cook Strait of New Zealand (41° 07′ S, 174° 27′ E) and supports a tuatara population of 500–550 individuals [30], [31]. The island is largely exposed rock, with low, scrubby vegetation on the northern face mainly composed of taupata (*Coprosma repens*) and horokaka (*Disphyma australe*) (Figure 1).

### Population surveys and assessment of body condition

Our methodology has been reported elsewhere for short population surveys over 3–5 nights [26] and a complete population census conducted in 2000 and 2001 over a combined 3 months of daily searches [31]. The dates, duration, and number of captures for each trip are reported in File S1 (Table S1 in File S1). Briefly, tuatara were captured by hand during nocturnal searches concentrated on the northern face of the island. Captured tuatara were given permanent, individual marks when first captured by toe-clipping prior to 2000 and by passive integrated transponders after 2001 (PIT tags; Allflex, ISO FDX-B, Dallas, Texas, USA). During each survey, animals were identified and measured (by the same researcher since 1991 for consistency, SNK). Temporary numbers were written on the side of each animal with nontoxic markers to identify of recaptures each night. Unmarked tuatara captured after the complete census in 2000 and 2001 were considered the next cohort maturing into adults. Juvenile tuatara are difficult to detect and are infrequently caught.

Trends in body condition across the period 1988–2001 were examined by Hoare et al. [30] and showed a significant decline in both sexes, with a more marked decline in female body condition. We examined changes in body condition with the inclusion of new data collected over the last decade using the same metric (the ratio of log-transformed mass to log-transformed snout-vent length). We used mixed-effects models to evaluate sex-specific differences over time in body condition. All models (including the null model) included sex, tail loss, and their interaction as fixed effects and individual as a random effect. We compared the null model with those including (1) time and (2) time and its interaction with sex (sex*time) using Akaike's information criterion (AIC). We computed Akaike weights (*w*) to identify the relative weight of evidence for each model conditional on the candidate model set. We then generated fitted estimates of body condition for each survey that accounted for individual variability and tail loss, based on the best fitting model.

### Ethics Statement

All appropriate ethics and protocol approvals were obtained for this research from the Victoria University of Wellington Animal Ethics Committee (approval number: 2012R33) and under permits from the New Zealand Department of Conservation (most recent permit number: NM-31919-FAU).

### Mark-recapture analysis

We estimated survival between 1988 and 2011 using open population mark-recapture models. The census methods in 2000 and 2001 prevented us from combining open and closed models in the robust design for this particular data set. Thus, we distilled the capture history for each year to a single record indicating capture or no capture for each individual. We excluded three surveys because the sampling was limited and the interval until the next survey was short (Table S1 in File S1). We also excluded the March 2012 survey because a male removal experiment was performed, increasing our detection of females during the survey (K. Grayson, *unpublished data*). The different procedure for this latter survey could not be accounted for by detection probability estimates, as the last survival and capture probabilities are confounded in a fully time-specific model [32].

We constructed a candidate model set testing all combinations of sex- and time-dependent survival, including either an interaction (sex*time) or additive effects (sex+time). We also modeled temporal variation in survival as a linear trend (*T*). Capture probability was modeled to include the effects of sex, time, sex+time, or as a constant (.). We tested for goodness-of-fit using the median ĉ approach to estimate overdispersion. Our most parameterized model fit the data well (ĉ = 1.02) and required no corrections for overdispersion. Akaike weights (*w*) were computed to identify the relative weight of evidence for each model conditional on the candidate model set [33].

### Predicting hatchling sex ratio

Field data on nesting and hatchling sex ratios are sparse and difficult to obtain due to the low number of gravid females each year, the cryptic nature of tuatara nesting, and the absence of secondary sexual characteristics in juveniles [31]. Thus, a model to predict the sex ratio of hatchlings on North Brother Island was developed by Mitchell et al. [34] using a spatially-explicit microclimate model to estimate hourly soil temperatures at typical nest depths, which were linked to a biophysical model to estimate the development rates and the sex of hatchlings. This approach used a high-resolution digital terrain model of North Brother Island to calculate the slope and aspect of known and potential nest sites. Surface and below ground temperatures at specified depths were predicted from a one-dimensional finite difference algorithm that simultaneously solves heat- and mass-balance equations, driven by averaged monthly climate data collated from a long term weather station on the island (maximum and minimum air temperatures, wind speeds, relative humidity, and cloud cover). Hourly soil temperature outputs were then used to calculate the development rate of embryos incubated at particular sites and depths, and the sex ratios were inferred from the constant temperature equivalent of the temperatures that fell within the thermosensitive period (the developmental time period when hatchling sex is determined) [34].

The published model [34] placed the timing of the thermosensitive period at the mid-point of development, based on the best information available at the time [28]. Here we update the hatchling sex ratio estimates based on more recent research that has clarified the timing of the thermosensitive period in tuatara using a temperature switching experiment [35]. We changed our model from placing the thermosensitive period at 50% of development to having sex determined at 35% of development and then recalculated the sex ratios estimated at typical nest locations at typical depths (50, 100, 150 and 200 mm), assuming that oviposition occurs in November or December. To assess the impacts of climate change on the hatchling sex ratios produced from these locations and nesting months, we adjusted the maximum and minimum monthly air temperature inputs in our model based on seasonally-projected increases compiled for Wellington (30 km east of North Brother Island) under a minimum and maximum warming scenario for 2080 [36]. These increases ranged from 0.1–0.8°C for the minimum warming scenario to 3.3–4°C for the maximum warming scenario.

### Population viability analysis

We performed population viability analyses using program VORTEX (ver. 9.99b) [37]. The initial life history parameters and their justification are detailed in Mitchell et al. [31]. For example, a baseline for adult survival of 95% for males and females was estimated by Nelson et al. [26] using survey data from 1988–1997. The annual reproductive rate of females was modeled as a density dependent function increasing from 15% of females breeding annually at high densities to 50% of females breeding annually at low densities, because both captive and wild females can breed every two years when resources are not limiting [38]. Due to the small size of the island, we did not incorporate an Allee effect under the assumption that females could still find mates even at low densities.

We systematically varied two input parameters: annual adult survival and a male bias in hatchling sex ratio. Adult survival was varied at equal 0.5% intervals for both males and females between 85% and 95%, whereas hatching sex ratios were varied between 50% and 100% males at increments of 2.5%. We quantified a sensitivity index for the effect of these parameters as S*e* = (Δ*e*/*e*)/(Δ*P*/*P*), where Δ*e*/*e* is the change in the extinction rate and Δ*P*/*P* is the proportional change in the parameter [39], [40]. We also compared viability under equal adult survival for males and females and with 1% reductions in female survival compared to males. We examined population viability over 2000 years (42–62 tuatara generations) in accordance with previous studies [31]. Longer timeframes are more appropriate in population viability analyses for species with long generation times [41], [42]. We simulated 500 populations for each combination of input parameters.

## Results

### Surveys and body condition

**Figure 2:**
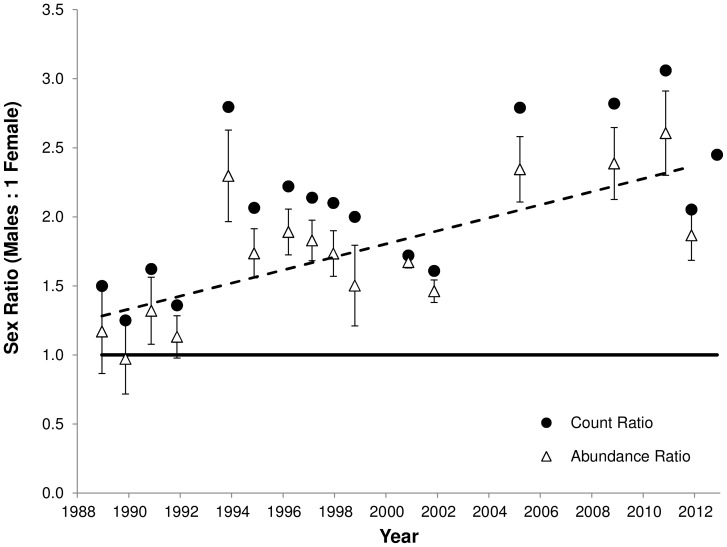
Temporal trends in tuatara population sex ratio on North Brother Island. We find an increasing male-biased trend in the adult sex ratio both using the count ratio directly from the number of individuals captured in each survey (1988–2012; black circles) and using an abundance ratio corrected for detection probability from the mark-recapture results (1988–2011; open triangles). The dashed line indicates the linear trend in the abundance ratio. Error bars on the abundance ratio data are ±1 SE calculated using the delta method.

The capture sex ratio for each trip is reported in File S1 (Table S1 in File S1). In accordance with Nelson et al. [26], we found 62.4% males in surveys from 1988–1998 (1.66 males:1 female). The results from the complete census in 2000 and 2001 are reported by Mitchell et al. [31], where a population sex ratio estimate of 60% males was found. Pooling individuals captured across surveys since then (2005–2012), we found a capture sex ratio of 70.3% males (2.36 males∶1 female). Newly captured individuals first encountered during this period since the complete census, which represent juveniles that have matured and developed secondary sexual characteristics, showed a similar male-bias with 69.9% males (2.32 males∶1 female). Survey-specific estimates of population sex ratio generated from the mark-recapture data and corrected for detection probability show a similar increase in the male sex ratio bias, with standard errors that do not overlap with a 1∶1 male:female ratio since 1994 (Figure 2).

**Figure 3:**
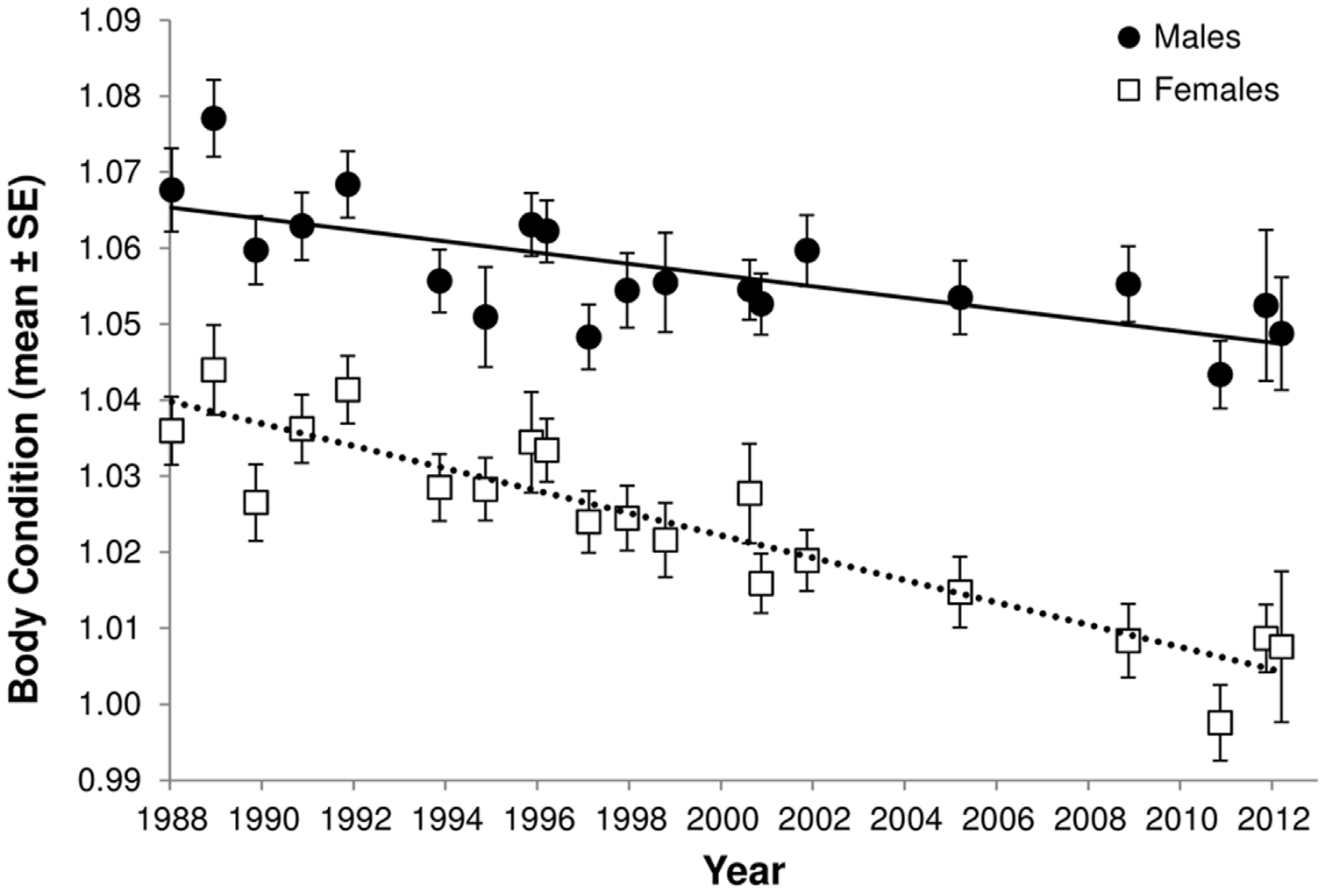
Body condition in adult male and female tuatara. Fitted body condition estimates (log mass/log snout-vent length ±1 SE) show declines for both male and female tuatara on North Brother Island from 1988 to 2012. The rate of decline for adult female body condition was found to be greater than the decline in adult males.

The decline in body condition first described by Hoare et al. [30] has continued over the last decade (Figure 3). We found overwhelming support for the model including an interaction between sex and time (weight of evidence  = 0.9999, ΔAIC_c_ for next best model without an interaction  = 19.8), with the rate of decline in body condition being 32% greater in females compared to males.

### Mark-recapture analysis

Our mark-recapture data set included 2773 captures of 621 individuals (400 males and 221 females) over the period January 1988 – November 2011. We compared 32 candidate models using an open population mark-recapture design to test the impact of sex and time on survival and capture probability. Three models garnered the majority of support (ΔAIC*_c_*≤2.10, cumulative weight of evidence  = 0.99 for the top three models; Table 1). All three modeled detection probability as an additive relationship between sex and time (sex+time). They also all included a linear trend in survival, and differed in the effects of sex on survival. All other capture probability structures had ΔAIC*_c_*>10 (Table S2 in File S1), indicating appreciably less support than for the top three models.

**Table 1:** Top model results from a candidate model set using tuatara survey data from 1998 – 2011 analyzed using an open mark-recapture population model.

Model Structure						
Survival	Capture Probability	AIC*_c_*	ΔAIC*_c_*	*K*	*w*	−2Log(L)
***T***	**sex + time**	**6195.3**	**0**	**19**	**0.490**	**6157.0**
***T*** ** + sex**	**sex + time**	**6196.1**	**0.79**	**20**	**0.331**	**6155.8**
***T*** * sex	**sex + time**	**6197.4**	**2.10**	**21**	**0.172**	**6155.1**
time	sex + time	6205.5	10.13	32	0.003	6140.6
sex + time	sex + time	6206.4	11.04	33	0.002	6139.5

Survival was modeled testing all combinations of sex- and time-dependent effects, including either an interaction (sex*time) or additive effects (sex+time). Temporal variation in survival was also modeled as a linear trend (*T*). Capture probability was modeled to include the effects of sex, time, sex+time, or constant. The top models gaining the majority of support are shown in bold. The rankings of the full model set can be found in File S1.

**Figure 4:**
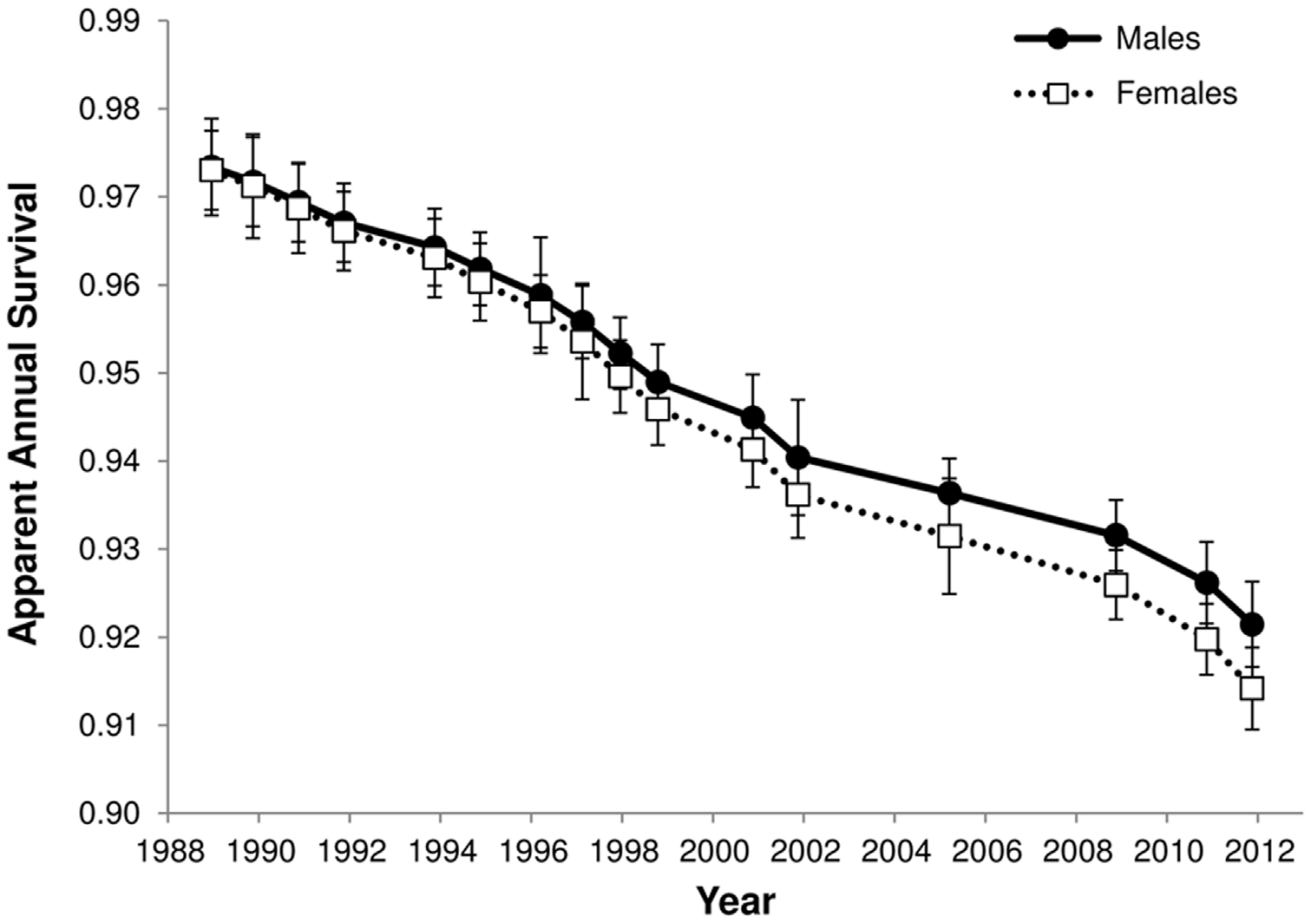
Annual survival estimates for male and female tuatara. Estimates were model averaged across the complete candidate model set based on the amount of support for each model. Estimates are shown ±1 SE.

We used model averaging to generate parameter estimates that accounted for the varying levels of support for each candidate model. Estimates of adult survival decreased over time (Figure 4). Survival for both sexes was estimated between 96–97% during the early years of the study and has declined to an estimate of 92.1%±0.01 SE for males and 91.4%±0.02 SE for females in 2011. As indicated by the inclusion of a sex effect in two of the three best models, there is evidence for separation between the sexes in survival, with lower estimates for females compared with males (Figure 4). Estimates of detection probability were highly variable across time, but the probability of detecting males was consistently 3–8% higher than the probability of detecting females.

### Predicting hatchling sex ratios

Our estimates of hatchling sex ratios produced from tuatara nests on North Brother Island are updated based on recent evaluation of the timing of the sex determining period during the development of tuatara embryos [35]. In essence, the major implication of this change is that sex is now determined in the model based on the thermal environment in late summer as opposed to autumn. This substantially reduces the percentage of nest sites predicted to produce all-female nests from 51.7% in Mitchell et al. [34] to 12.5%, and we now predict that most nest sites that are currently used by tuatara on North Brother Island would produce mixed sexes (Table 2). We now estimate that typical nest sites on North Brother Island overall produce 56% males (1.27 males∶ 1 female) under the recent averaged climate. The new estimate is more similar to the small amount of sex ratio data available for natural nests on North Brother Island [31]. Applying projected regional increases in air temperature to the updated model results in very similar sex ratio predictions to those previously reported [34]. Under the minimum climate change scenario (0.1–0.8°C air temperature increase by 2080) the predicted hatchling sex ratio shifts slightly to 57% males, but if air temperatures increase by 3.3–4°C by 2080 based on projections of maximum warming scenarios for New Zealand, then current nest sites would produce entirely male hatchlings (Table 2).

**Table 2:** Proportions of tuatara nest types and the aggregate hatchling sex ratio predicted at current nesting locations under current and future climates.

	Current Climate	Minimum Warming 2080	Maximum Warming 2080
All-female nests (%)	12.5	11.5	0
Mixed-sex nests (%)	62.5	62.3	0
All-male nests (%)	25.0	26.2	100
Proportion Males	0.56	0.57	1.0

Oviposition occurs in early November or early December. These predictions update those previously reported, due to new information on the timing of the thermosensitive period for sex determination in tuatara, which was previously thought to fall at 50% of development, but is now known to occur at 35% of embryonic development.

### Population viability analysis

**Figure 5:**
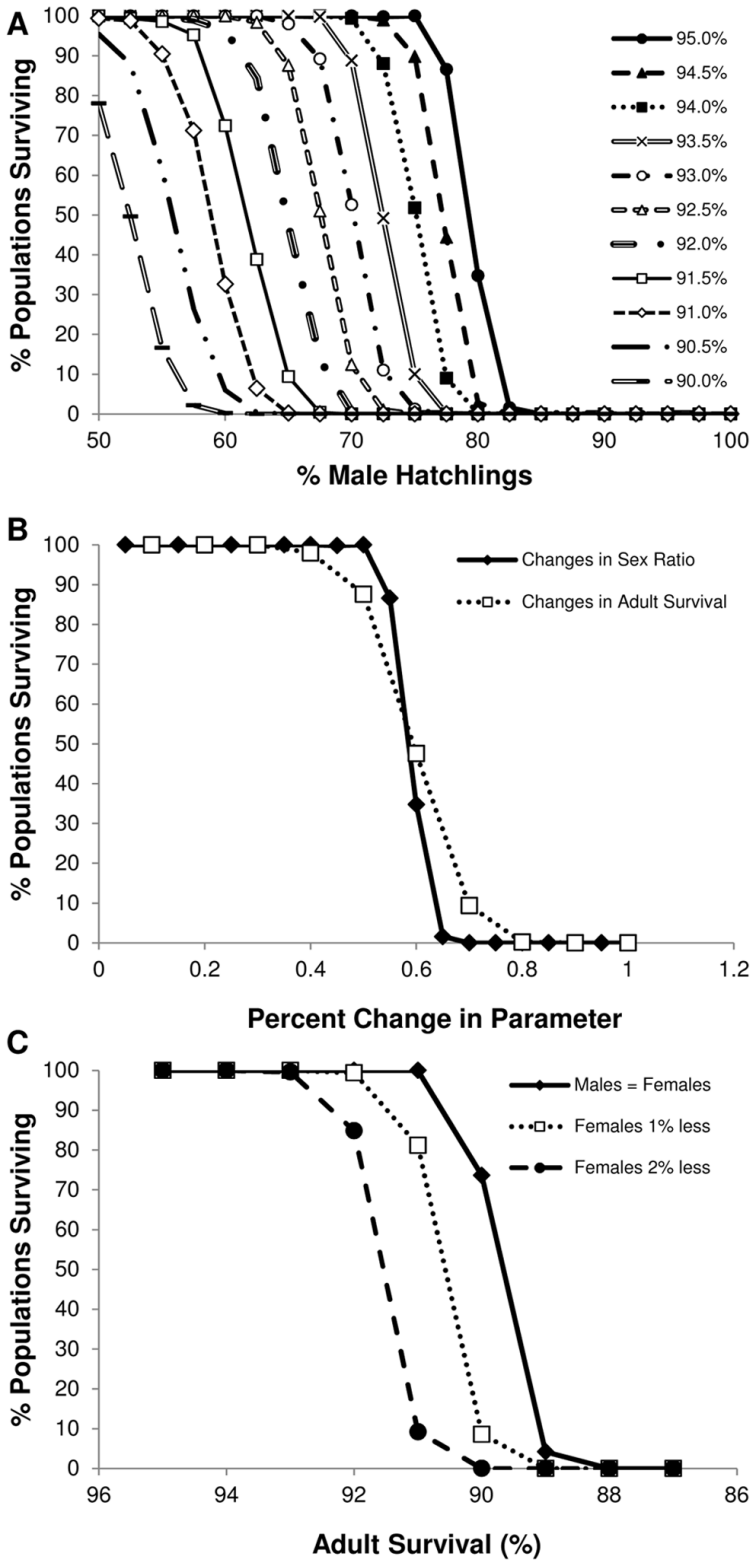
Extinction probability for tuatara on North Brother Island. Viability is assessed by the percentage of simulated populations predicted to remain after 2000 years (50–64 tuatara generations). Population extinction occurs when only one sex remains. A) The relationship between decreases in adult survival and increases in the male-bias of hatchling sex ratios. B) The sensitivity of percentage changes in hatchling sex ratio and adult survival. Data on changes in hatchling sex ratio are shown at a constant 95% adult survival and changes in adult survival are shown at a constant 65% male-biased hatchling sex ratio. C) The effect on viability with reductions in adult female survival compared to males, using a constant equal sex ratio of hatchlings.

When examining changes in a single vital rate from the baseline scenario, the tuatara population on North Brother Island is predicted to persist at hatchling sex ratios of up to 75% males and reductions in adult survival down to 91%. Extinction probability becomes 100% at a hatchling sex ratio of 85% males (mean time to extinction  = 388.2 years ±8.8 SE) and adult survival of 88% (mean time to extinction  = 364.8 years ±7.6 SE). Together, these factors have an additive effect on extinction risk (Figure 5A). For example, at 93% adult survival the population only remains viable at hatchling sex ratios of up to 65% males. Our analyses indicate that adult survival is less sensitive and has a more gradual effect on extinction risk relative to changes in hatching sex ratio (S*e* = 2.5±0.8 SE for adult survival and S*e* = 5.0±2.2 SE for hatchling sex ratio; Figure 5B). Reduced female survival compared to that of males also decreases population viability. Under equal survival rates between the sexes, populations remain viable until adult survival drops below 91%. With female survival reduced an additional 1% and 2% less than males, viability declines at 92% and 93% adult male survival. In other words, additional decreases in female survival relative to males changes the adult survival limit for viability by an equivalent percentage (Figure 5C).

Our mark-recapture results and updated model of hatching sex ratios indicate that the population has already deviated from a baseline scenario of an equal hatchling sex ratio and similar survival between the sexes. Our simulations using parameters from the current population (female survival  = 91%±2.4 SD, male survival  = 92%±2.2 SD, 56% males at hatching) result in a 12% probability of extinction within the 2000 year timeframe of the analysis (60 of 500 simulated populations become extinct, mean time to extinction  = 1183.3 years ±59.5 SE).

## Discussion

**Figure 6:**
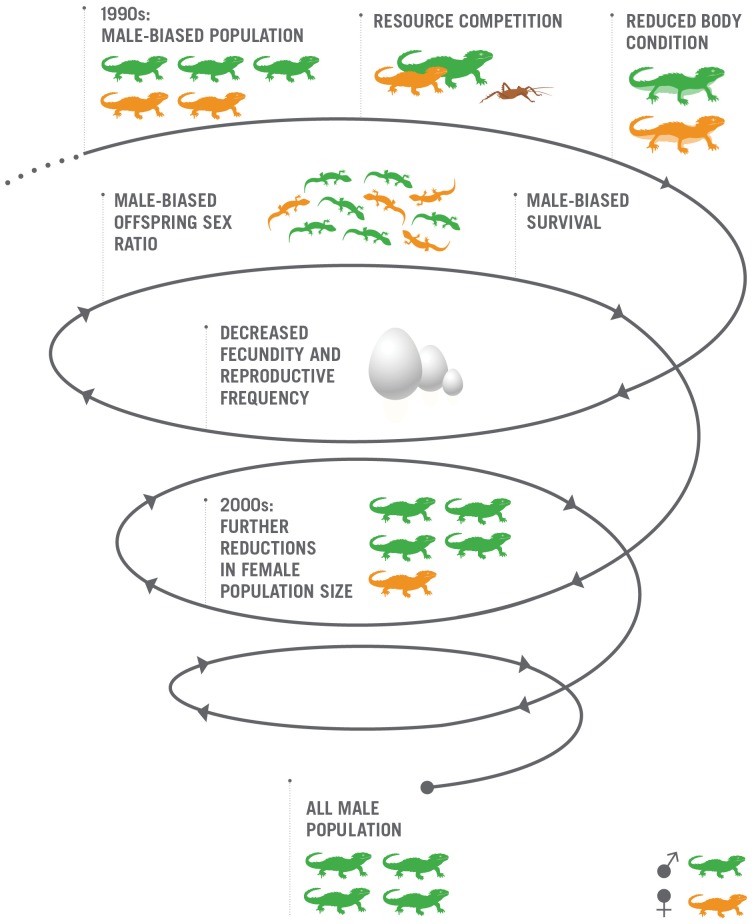
The extinction vortex in North Brother tuatara. The cumulative feedbacks from a male-biased sex ratio are reducing female numbers and driving an island population of tuatara towards becoming entirely composed of males and functionally extinct. Graphic designed by C. Foster.

Our survey and mark-recapture results confirm that the male bias in the adult tuatara population on North Brother Island is increasing. We find strong support for three compounding factors (decreased female body condition, reduced survival, and a male-biased hatchling sex ratio) contributing to a downward spiral in population viability, creating the potential for an extinction vortex (Figure 6). Our data show that a likely result of further increases in the male-biased sex ratio on North Brother Island is a functionally extinct, all-male population. This extinction scenario appears to be unique to the tuatara population on North Brother Island. We have not observed a sex ratio bias in the other closely monitored population on Stephens Island [43], a 150 ha island also in the Cook Strait with a substantially larger tuatara population and variety of habitats.

We find the trend of declining body condition continuing over the past decade in both sexes, with a more pronounced decline in females. Male tuatara are larger than females, and are territorial and socially dominant [43], [44]. Experimental work in juveniles has shown that aggressive behavior is related to body size, and under conditions of resource competition males outcompete females for food [45]. Dominance by large males is also found in the social system of many lizard species [46]. Male harassment can play a role in reducing the condition of females and can intensify under male-biased sex ratios [21], [47].

Reduced body condition can have direct impacts on female fitness. Two separate studies have found severely reduced fecundity in female tuatara on North Brother Island, with females breeding approximately every nine years [31], [48] compared to every 2–5 years in tuatara on nearby Stephens Island [27]. Declines in female body condition can have compounding impacts on the number and quality of offspring [49]. Reduced body condition also contributes to the next component of the extinction vortex: decreased survival. In addition to overall reductions in annual adult survival, the rankings in our mark-recapture model indicate that sex-based differences in survival are becoming more important for explaining trends in the data. The decline in survival may be an early indication of overall population decline, or may reflect changes in environmental conditions that have been less favorable for adult tuatara. While field captures of juveniles on North Brother Island are too sparse to provide information on their survival, we expect that the male-biased sex ratio and resource competition have the same outcome for juveniles as for adult females, for two reasons. First, survival of juvenile females was 10% lower compared to juvenile males after hatching over five years in semi-natural conditions at a tuatara head-starting facility on the New Zealand mainland [50]. Second, we found a male bias in newly captured tuatara over the last decade, a cohort that likely represents juveniles reaching maturity.

Our capture data and mark-recapture results indicate a continued decrease in the female tuatara population on North Brother Island. Previous studies using data from 1988–2001 reported population sex ratio estimates of 60–62% males [26], [31]. Since these studies, we have seen an increasing trend in the male bias with captures over the last decade averaging above 70% males. A challenge for mark-recapture studies with cryptic species is that all animals are not available for capture [51]. Tuatara are burrow-dwelling and have weather-dependent activity and it is likely that many individuals cannot be detected over an entire survey period. If the undetectable portion of the population was consistently biased towards females, our results could over-estimate the number of males. However, we believe our long-term data, consistency in survey methods, and mark-recapture methods that account for detection probability address this concern.

The final component of the extinction vortex is the male-biased hatchling sex ratio. Species with TSD can be susceptible to demographic shifts due to changes in habitat, alteration of the thermal environment, or stochasticity at small population sizes due to the nesting behavior of a few individuals. Global climate change poses a unique risk to the hatchling sex ratios of species with TSD [52]; however, the TSD pattern present in the majority of reptile species produces females at warmer temperatures, which may pose less of a threat to population viability or even enhance it in the short-term [14], [16]. The female-to-male pattern of sex determination in tuatara places the species at particular risk from rapidly increasing air temperatures [14], [53]. Our updated biophysical model indicates that the thermal conditions in the available nesting habitats are likely to produce slightly male-biased hatchling sex ratios in tuatara on North Brother Island under the recent climate. The stark contrast in predictions from the minimum and maximum warming scenarios (57% vs. 100% male hatchlings, respectively) reflects the narrow transitional range between temperatures for the production of females compared with males in the TSD pattern for tuatara [28]. The present trend in temperature increases for New Zealand, without future changes in greenhouse gas emissions, align closely with projections from a maximum warming scenario [54]. This indicates that production of all-male hatchlings in the near future is not just a worst-case scenario, but a real possibility.

The potential for altering this scenario in the current population includes management intervention, an evolutionary response, or behavioral plasticity. Management actions could target sex ratios through artificial incubation, head starting juvenile females, or translocations of adults [31], [55]. However, manipulation of sex ratios and management by supplementing females or harvesting males might offer a short-term solution, but may not improve long-term viability without sustained effort. Adaptive responses include plasticity or microevolution of maternal nesting behaviour or the pivotal temperature for sex determination [14], [56], [57]. The long evolutionary history of Sphenodontian reptiles dating back over 200 million years suggests that the ancestors of modern day tuatara have persisted through major shifts in climate; however, we do not know the effects that past climatic changes had on particular populations or the distribution of species in the lineage. Regardless, the capacity for tuatara on North Brother Island to reverse the male-bias in the population is limited. Rapid evolution of the pivotal temperature is unlikely due to the low genetic diversity in the population and the long generation time [31], [58]. Changes in nesting phenology or habitat might allow female tuatara to avoid more extreme nest temperatures and to shift the hatchling sex ratio but nesting habitat on North Brother Island is limited, particularly since human modification in the 1800s, and offers very few locations where cooler nest temperatures could be realized [34]. Adaptive responses to changing climates that were possible when the species occupied the entire mainland of New Zealand, such as migration to a different climate or habitat, immigration of females or new genetic stock, or emigration of excess males, would all require management intervention for an island population. Additionally, the pace of current climate change is exceeding rates of historical climate change [59], [60].

Under current vital rates, the risk of extinction for this population appears low and far into the future. However, our modeling suggests that slight further declines in adult survival and the proportion of female hatchlings, alone or in concert, threaten this population with extinction. Several other factors not included in our population viability model also increase the risk of population extinction. It is likely that juveniles are also experiencing declines in survival and a disparity in survival between the sexes. Measurements of fecundity and reproductive frequency on North Brother Island were performed 1–2 decades ago [31], [48] and further declines in fecundity are possible. Inbreeding depression has been modeled previously and only worsens the population outlook [14]. Thus, we expect that our viability models are conservative. However, the population could numerically persist for over a hundred years even with an all-male population, due to the extreme longevity of tuatara (known to be at least 70 yrs in the wild [61], and increasing along with the duration of our studies).

Taken together, the mechanisms we outline paint a grim outlook for female tuatara on North Brother Island, with little opportunity for change in the population trajectory without intervention. Potential interventions, such as translocations, habitat modifications, or artificially incubating eggs, need to be carefully considered before implementation. An alternative is to not intervene, and to value this population as a case study of the capacity of isolated populations to adapt to climate change and sex ratio bias. A recent review of bird species indicates that sex ratio biases may be more common than expected, particularly in threatened populations [6]. The potential for population sex ratio to fluctuate and the resulting impacts to demographic parameters can depend on the ecology and behavior of a species [62]. For species at risk from sex ratio skew, we have demonstrated here that a reasonable number of individuals in a population may not equate to a viable population if feedbacks are operating to exacerbate the bias. In the case of North Brother tuatara, these impacts are occurring over decades due to the extremely slow life history of the species. In other species, changes in vital rates and population composition can happen much more quickly. Isolated and small populations in particular, already well-known to conservation managers as being at higher risk of extinction, are more susceptible to the compounding negative effects from a male-biased sex ratio on population growth.

## Supporting Information

File S1 Table S1, Complete tuatara capture numbers for North Brother Island surveys from January 1988 – March 2012. Table S2, Model selection results for the complete candidate model set using tuatara survey data from 1998 – 2011 analyzed using an open mark-recapture population model.
(DOCX)

## References

[1] Wedekind C (2002) Manipulating sex ratios for conservation: short-term risks and long-term benefits. Anim Conserv 5: 13–20.

[2] Dale S (2001) Female-biased dispersal, low female recruitment, unpaired males, and the extinction of small and isolated bird populations. Oikos 92: 344–356.

[3] López-Sepulcre A, Norris K, Kokko H (2009) Reproductive conflict delays the recovery of an endangered social species. J Anim Ecol 78: 219–225. 18811660 10.1111/j.1365-2656.2008.01475.x

[4] Clout MN, Elliott GP, Robertson BC (2002) Effects of supplementary feeding on the offspring sex ratio of kakapo: a dilemma for the conservation of a polygynous parrot. Biol Conserv 107: 13–18.

[5] Lande R (1993) Risks of population extinction from demographic and environmental stochasticity and random catastrophes. Am Nat 142: 911–927. 29519140 10.1086/285580

[6] Donald PF (2007) Adult sex ratios in wild bird populations. Ibis 149: 671–692.

[7] Ginsberg JR, Milner-Gulland EJ (1994) Sex-biased harvesting and population dynamics in ungulates: implications for conservation and sustainable use. Conserv Biol 8: 157–166.

[8] Trivers RL, Willard DE (1973) Natural selection of parental ability to vary the sex ratio of offspring. Science 179: 90–92. 4682135 10.1126/science.179.4068.90

[9] Nager R, Monaghan P, Griffiths R, Houston D, Dawson R (1999) Experimental demonstration that offspring sex ratio varies with maternal condition. Proc Natl Acad Sci USA 96: 570–573. 9892674 10.1073/pnas.96.2.570 PMC15177

[10] Robertson BC, Elliott GP, Eason DK, Clout MN, Gemmell NJ (2006) Sex allocation theory aids species conservation. Biol Lett 2: 229–231. 17148369 10.1098/rsbl.2005.0430 PMC1618899

[11] Freed LA, Cann RL, Diller K (2009) Sexual dimorphism and the evolution of seasonal variation in sex allocation in the Hawaii akepa. Evol Ecol Res 11: 731–757.

[12] Korpelainen H (1990) Sex ratios and conditions required for environmental sex determination in animals. Biol Rev 65: 147–184. 2190635 10.1111/j.1469-185x.1990.tb01187.x

[13] Bull JJ (1980) Sex determination in reptiles. Q Rev Biol 55: 3–21.

[14] Mitchell NJ, Janzen FJ (2010) Temperature-dependent sex determination and contemporary climate change. Sex Dev 4: 129–140. 20145383 10.1159/000282494

[15] Mrosovsky N (1982) Sex ratio bias in hatchling sea turtles from artificially incubated eggs. Biol Conserv 23: 309–314.

[16] Wapstra E, Uller T, Sinn DL, Olsson M, Mazurek K, et al (2009) Climate effects on offspring sex ratio in a viviparous lizard. J Anim Ecol 78: 84–90. 18811661 10.1111/j.1365-2656.2008.01470.x

[17] Freedberg S, Bowne DR (2006) Monitoring juveniles across years reveals non-Fisherian sex ratios in a reptile with environmental sex determination. Evol Ecol Res 8: 1499–1510.

[18] Valenzuela N, Lance VA (2004) Temperature-Dependent Sex Determination in Vertebrates: Smithsonian Books, Washington, DC.

[19] Kvarnemo C, Ahnesjo I (1996) The dynamics of operational sex ratios and competition for mates. Trends Ecol Evol 11: 404–408. 21237898 10.1016/0169-5347(96)10056-2

[20] Arnqvist G, Rowe L (2005) Sexual Conflict. Princeton, New Jersey: Princeton University Press.

[21] Le Galliard JF, Fitze PS, Ferrière R, Clobert J (2005) Sex ratio bias, male aggression, and population collapse in lizards. Proc Natl Acad Sci U S A 102: 18231–18236. 16322105 10.1073/pnas.0505172102 PMC1312374

[22] Rankin DJ, Dieckmann U, Kokko H (2011) Sexual conflict and the tragedy of the commons. Am Nat 177: 780–791. 21597254 10.1086/659947

[23] Gilpin ME, Soule ME (1986) Minimum viable populations: processes of species extinction. In: Soule ME, editor. Conservation Biology: The Science of Scarcity and Diversity. Sunderland, MA: Sinauer Associates. pp. 19–34.

[24] Brook BW, Tonkyn DW, O'Grady JJ, Frankham R (2002) Contribution of inbreeding to extinction risk in threatened species. Conserv Ecol 6: 16.

[25] Stephens PA, Sutherland WJ (1999) Consequences of the Allee effect for behaviour, ecology and conservation. Trends Ecol Evol 14: 401–405. 10481204 10.1016/s0169-5347(99)01684-5

[26] Nelson NJ, Keall SN, Pledger S, Daugherty CH (2002) Male-biased sex ratio in a small tuatara population. J Biogeogr 29: 633–640.

[27] Cree A (1994) Low annual reproductive output in female reptiles from New Zealand. N Z J Zool 21: 351–372.

[28] Mitchell NJ, Nelson NJ, Cree A, Pledger S, Keall SN, et al (2006) Support for a rare pattern of temperature-dependent sex determination in archaic reptiles: evidence from two species of tuatara (*Sphenodon*). Front Zool 3: 9. 16808840 10.1186/1742-9994-3-9 PMC1559618

[29] Gaze P (2001) Tuatara Recovery Plan 2001–2011. Wellington, New Zealand: Biodiversity Recovery Unit, New Zealand Department of Conservation.

[30] Hoare JM, Pledger S, Keall SN, Nelson NJ, Mitchell NJ, et al (2006) Conservation implications of a long-term decline in body condition of the Brothers Island tuatara (*Sphenodon guntheri*). Anim Conserv 9: 456–462.

[31] Mitchell NJ, Allendorf FW, Keall SN, Daugherty CH, Nelson NJ (2010) Demographic effects of temperature-dependent sex determination: will tuatara survive global warming? Glob Chang Biol 16: 60–72.

[32] Lebreton JD, Burnham KP, Clobert J, Anderson DR (1992) Modeling survival and testing biological hypotheses using marked animals: a unified approach with case studies. Ecol Monogr 62: 67–118.

[33] Burnham KP, Anderson DR (2002) Model Selection and Multimodel Inference: A Practical Information-Theoretic Approach. New York, USA: Springer Verlag.

[34] Mitchell NJ, Kearney MR, Nelson NJ, Porter WP (2008) Predicting the fate of a living fossil: how will global warming affect sex determination and hatching phenology in tuatara? Proc R Soc Lond B Biol Sci 275: 2185–2193. 10.1098/rspb.2008.0438 PMC2603239 18595840

[35] Nelson NJ, Moore JA, Pillai S, Keall SN (2010) Thermosensitive period for sex determination. Herpetol Conserv Biol 5: 324–329.

[36] Wratt DS, Mullan AB, Salinger MJ, Allan S, Morgan T, et al. (2004) Climate Change Effects and Impacts Assessment: A Guidance Manual for Local Government in New Zealand. Wellington, New Zealand: New Zealand Climate Change Office, Ministry for the Environment.

[37] Lacy RC (1993) VORTEX: a computer simulation model for population viability analysis. Wildl Res 20: 45–65.

[38] Moore JA, Nelson NJ, Keall SN, Daugherty CH (2008) Implications of social dominance and multiple paternity for the genetic diversity of a captive-bred reptile population (tuatara). Conserv Genet 9: 1243–1251.

[39] Pulliam HR, Dunning JB Jr, Liu J (1992) Population dynamics in complex landscapes: a case study. Ecol Appl 2: 165–177. 27759202 10.2307/1941773

[40] Hu J, Jiang Z, Mallon DP (2013) Metapopulation viability of a globally endangered gazelle on the Northeast Qinghai-Tibetan Plateau. Biol Conserv 166: 23–32.

[41] Armbruster P, Fernando P, Lande R (1999) Time frames for population viability analysis of species with long generations: an example with Asian elephants. Anim Conserv 2: 69–73.

[42] O'Grady JJ, Reed DH, Brook BW, Frankham R (2008) Extinction risk scales better to generations than to years. Anim Conserv 11: 442–451.

[43] Moore JA, Daugherty CH, Nelson NJ (2009) Large male advantage: phenotypic and genetic correlates of territoriality in tuatara. J Herpetol 43: 570–578.

[44] Herrel A, Moore JA, Bredeweg EM, Nelson NJ (2010) Sexual dimorphism, body size, bite force and male mating success in tuatara. Biol J Linn Soc 100: 287–292.

[45] Woerner LLB (2009) Aggression and competition for space and food in captive juvenile tuatara (*Sphenodon punctatus*). Wellington, New Zealand: Victoria University of Wellington.

[46] Alberts AC (1994) Dominance hierarchies in male lizards: Implications for zoo management programs. Zoo Biol 13: 479–490.

[47] Clutton-Brock TH, Parker GA (1995) Sexual coercion in animal societies. Anim Behav 49: 1345–1365.

[48] Cree A, Daugherty CH, Schafer SF, Brown D (1991) Nesting and clutch size of tuatara (*Sphenodon guntheri*) on North Brother Island, Cook Strait. Tuatara 31: 9–16.

[49] Bernardo J (1996) Maternal effects in animal ecology. Am Zool 36: 83–105.

[50] Gruber MAM (2007) Conservation of tuatara (*Sphenodon)*: an evaluation of the survival and growth of artificially incubated, head-started juveniles. Wellington, New Zealand: Victoria University of Wellington.

[51] Kendall WL, Nichols JD, Hines JE (1997) Estimating temporary emigration using capture-recapture data with Pollock's robust design. Ecology 78: 563–578.

[52] Janzen FJ (1994) Climate change and temperature-dependent sex determination in reptiles. Proc Natl Acad Sci U S A 91: 7487–7490. 8052608 10.1073/pnas.91.16.7487 PMC44426

[53] Nelson NJ, Thompson MB, Pledger S, Keall SN, Daugherty CH (2004) Do TSD, sex ratios, and nest characteristics influence the vulnerability of tuatara to global warming? Int Congr Ser 1275: 250–257.

[54] Reisinger A, Mullan B, Manning M, Wratt DS, Nottage RAC (2010) Global and local climate change scenarios to support adaptation in New Zealand. Wellington, New Zealand: New Zealand Climate Change Centre.

[55] Miller KA, Miller HC, Moore JA, Mitchell NJ, Cree A, et al (2012) Securing the demographic and genetic future of tuatara through assisted colonization. Conserv Biol 26: 790–798. 22827440 10.1111/j.1523-1739.2012.01902.x

[56] Doody JS, Guarino E, Georges A, Corey B, Murray G, et al (2006) Nest site choice compensates for climate effects on sex ratios in a lizard with environmental sex determination. Evol Ecol 20: 307–330.

[57] Schwanz LE, Janzen FJ (2008) Climate change and temperature-dependent sex determination: can individual plasticity in nesting phenology prevent extreme sex ratios? Physiol Biochem Zool 81: 826–834. 18831689 10.1086/590220

[58] MacAvoy ES, McGibbon LM, Sainsbury JP, Lawrence H, Wilson CA, et al (2007) Genetic variation in island populations of tuatara (*Sphenodon* spp) inferred from microsatellite markers. Conserv Genet 8: 305–318.

[59] Marcott SA, Shakun JD, Clark PU, Mix AC (2013) A reconstruction of regional and global temperature for the past 11,300 years. Science 339: 1198–1201. 23471405 10.1126/science.1228026

[60] Mann ME, Zhang Z, Hughes MK, Bradley RS, Miller SK, et al (2008) Proxy-based reconstructions of hemispheric and global surface temperature variations over the past two millennia. Proc Natl Acad Sci U S A 105: 13252–13257. 18765811 10.1073/pnas.0805721105 PMC2527990

[61] Thompson MB, Daugherty CH, Cree A, French DC, Gillingham JC, et al (1992) Status and longevity of the tuatara, *Sphenodon guntheri*, and Duvaucel's gecko, *Hoplodactylus duvaucelii*, on North Brother Island, New Zealand. J R Soc N Z 22: 123–130.

[62] Ewen JG, Thorogood R, Armstrong DP (2011) Demographic consequences of adult sex ratio in a reintroduced hihi population. J Anim Ecol 80: 448–455. 21083871 10.1111/j.1365-2656.2010.01774.x

