# Supplementary material for: Sex Ratio Bias and Extinction Risk in an Isolated Population of Tuatara (Sphenodon punctatus)
Source: PLoS One. 2014 Apr 8;9(4):e94214. doi: 10.1371/journal.pone.0094214 (PMC3979778; doi:10.1371/journal.pone.0094214)
Supplement: File S1 — Table S1, Complete tuatara capture numbers for North Brother Island surveys from January 1988 – March 2012. Table S2, Model selection results for the complete candidate model set using tuatara survey data from 1998 – 2011 analyzed using an open mark-recapture population model. (DOCX) [file pone.0094214.s001.docx]

**Supporting Information:**

Sex ratio bias and extinction risk in an isolated population of tuatara (*Sphenodon punctatus*)

Kristine L. Grayson, Nicola J. Mitchell, Joanne M. Monks, Susan N. Keall, Joanna N. Wilson, Nicola J. Nelson

**Table S1**. Summary of tuatara capture data on North Brother Island 1988 – 2012.

| Trip No. | Date | Survey Nights | Juveniles Captured | Adults Captured | Sex Ratio  (Males:1 Female) | % Male | Used in Open Population Model |
| --- | --- | --- | --- | --- | --- | --- | --- |
| 1 | Jan 88 | 3 | 1 | 99 | 2.09 | 67.7 | ✓ |
| 2 | Dec 88 | 3 | 0 | 30 | 1.50 | 60.0 | ✓ |
| 3 | Nov 89 | 3 | 0 | 36 | 1.25 | 55.6 | ✓ |
| 4 | Oct 90 | 7 | 0 | 51 | 1.55 | 60.8 |  |
| 5 | Nov 90 | 3 | 0 | 97 | 1.62 | 61.9 | ✓ |
| 6 | Nov 91 | 3 | 0 | 92 | 1.36 | 57.6 | ✓ |
| 7 | Nov 93 | 5 | 2 | 167 | 2.80 | 73.7 | ✓ |
| 8 | Nov 94 | 3 | 1 | 187 | 2.07 | 67.4 | ✓ |
| 9 | Nov 95 | 1 | 0 | 36 | 1.40 | 58.3 |  |
| 10 | Mar 96 | 5 | 2 | 219 | 2.22 | 68.9 | ✓ |
| 11 | Feb 97 | 5 | 3 | 226 | 2.14 | 68.1 | ✓ |
| 12 | Dec 97 | 5 | 2 | 186 | 2.10 | 67.7 | ✓ |
| 13 | Oct 98 | 2 | 0 | 51 | 2.00 | 66.7 | ✓ |
| 14 | Aug 00 | 1 | 0 | 34 | 2.09 | 67.6 |  |
| 15 | Nov-Dec 2000 | 52 | 4 | 359 | 1.72 | 63.2 | ✓ |
| 16 | Nov-Dec 2001 | 32 | 1 | 266 | 1.61 | 61.7 | ✓ |
| 17 | Mar 05 | 5 | 1 | 163 | 2.79 | 73.6 | ✓ |
| 18 | Nov 08 | 4 | 3 | 149 | 2.82 | 73.8 | ✓ |
| 19 | Nov 10 | 5 | 1 | 134 | 3.06 | 75.4 | ✓ |
| 20 | Nov 11 | 5 | 0 | 174 | 2.05 | 67.2 | ✓ |
| 21 | Mar 12 | 4 | 1 | 138 | 2.45 | 71.0 | ✓ |

**Table S2.** Model selection results for candidate models using tuatara survey data from 1998 – 2011 analyzed using an open mark-recapture population model. Survival was modeled testing all combinations of sex- and time-dependent effects, including either an interaction (sex*time) or additive effects (sex+time). Temporal variation in survival was also modeled as a linear trend (*T*). Capture probability was modeled to include the effects of sex, time, sex+time, or constant (.). The top models gaining the majority of support are shown in bold.

| Model Structure | | |  |  |  |  |  |
| --- | --- | --- | --- | --- | --- | --- | --- |
| Survival | Capture Probability | | AIC*_c_* | ΔAIC*_c_* | *K* | *w* | -2Log(L) |
| ***T*** | **sex + time** | **6195.3** | | **0** | **19** | **0.49** | **6157.0** |
| ***T* + sex** | **sex + time** | **6196.1** | | **0.79** | **20** | **0.33** | **6155.8** |
| ***T* * sex** | **sex + time** | **6197.4** | | **2.10** | **21** | **0.17** | **6155.1** |
| time | sex + time | 6205.5 | | 10.13 | 32 | 0.003 | 6140.6 |
| sex + time | sex + time | 6206.4 | | 11.04 | 33 | 0.002 | 6139.5 |
| . | sex + time | 6208.9 | | 13.52 | 18 | 0.0006 | 6172.6 |
| *T* + sex | time | 6209.6 | | 14.22 | 19 | 0.0004 | 6171.3 |
| sex | sex + time | 6210.0 | | 14.63 | 19 | 0.0003 | 6171.7 |
| *T* | time | 6210.6 | | 15.25 | 18 | 0.0002 | 6174.3 |
| *T* * sex | time | 6210.8 | | 15.46 | 20 | 0.0002 | 6170.5 |
| sex + time | time | 6220.5 | | 25.20 | 32 | 0 | 6155.7 |
| time | time | 6221.4 | | 26.06 | 31 | 0 | 6158.6 |
| sex * time | sex + time | 6222.8 | | 27.44 | 48 | 0 | 6124.8 |
| sex | time | 6223.7 | | 28.31 | 18 | 0 | 6187.4 |
| . | time | 6224.7 | | 29.34 | 17 | 0 | 6190.4 |
| sex * time | time | 6235.5 | | 40.13 | 47 | 0 | 6139.6 |
| time | sex | 6791.6 | | 596.28 | 18 | 0 | 6755.3 |
| sex + time | sex | 6791.8 | | 596.44 | 19 | 0 | 6753.5 |
| sex + time | . | 6801.5 | | 606.19 | 18 | 0 | 6765.2 |
| time | . | 6803.7 | | 608.34 | 17 | 0 | 6769.4 |
| sex * time | sex | 6807.1 | | 611.73 | 34 | 0 | 6738.1 |
| sex * time | . | 6818.4 | | 623.09 | 33 | 0 | 6751.5 |
| *T* | sex | 6837.1 | | 641.72 | 4 | 0 | 6829.0 |
| *T* + sex | sex | 6837.9 | | 642.55 | 5 | 0 | 6827.9 |
| *T* * sex | sex | 6838.4 | | 643.10 | 6 | 0 | 6826.4 |
| . | sex | 6841.4 | | 646.02 | 3 | 0 | 6835.4 |
| sex | sex | 6842.4 | | 647.04 | 4 | 0 | 6834.4 |
| *T* + sex | . | 6849.0 | | 653.65 | 4 | 0 | 6841.0 |
| *T* * sex | . | 6849.3 | | 653.94 | 5 | 0 | 6839.3 |
| *T* | . | 6850.6 | | 655.24 | 3 | 0 | 6844.6 |
| sex | . | 6853.5 | | 658.12 | 3 | 0 | 6847.5 |
| . | . | 6854.9 | | 659.51 | 2 | 0 | 6850.8 |
